# Three-Layer PdO/CuWO_4_/CuO System for Hydrogen Gas Sensing with Reduced Humidity Interference

**DOI:** 10.3390/nano11123456

**Published:** 2021-12-20

**Authors:** Nirmal Kumar, Stanislav Haviar, Petr Zeman

**Affiliations:** Department of Physics and NTIS—European Centre of Excellence, Faculty of Applied Sciences, University of West Bohemia, Pilsen 301 00, Czech Republic; kumarn@kfy.zcu.cz (N.K.); zemanp@kfy.zcu.cz (P.Z.)

**Keywords:** conductometric hydrogen sensor, copper tungstate, CuWO_4_, reactive sputtering, nanoheterojunction

## Abstract

The growing hydrogen industry is stimulating an ongoing search for new materials not only for hydrogen production or storage but also for hydrogen sensing. These materials have to be sensitive to hydrogen, but additionally, their synthesis should be compatible with the microcircuit industry to enable seamless integration into various devices. In addition, the interference of air humidity remains an issue for hydrogen sensing materials. We approach these challenges using conventional reactive sputter deposition. Using three consequential processes, we synthesized multilayer structures. A basic two-layer system composed of a base layer of cupric oxide (CuO) overlayered with a nanostructured copper tungstate (CuWO_4_) exhibits higher sensitivity than individual materials. This is explained by the formation of microscopic heterojunctions. The addition of a third layer of palladium oxide (PdO) in forms of thin film and particles resulted in a reduction in humidity interference. As a result, a sensing three-layer system working at 150 °C with an equalized response in dry/humid air was developed.

## 1. Introduction

As a prominent clean source of energy, hydrogen plays an important role in power generation. Its vast abundance, non-toxic nature, and high combustion efficiency make it a promising source of energy in the future. The highly flammable, bland, odorless, and colorless character and wide explosive concentration of hydrogen demand the development of sensors and detectors. Among many approaches, one of the most common are chemiresistors based on metal oxide semiconductors (MOS) [1,2,3,4].

Primarily, binary oxides such as WO_3_, TiO_2_, SnO_2_, CuO, NiO, etc., have been investigated as chemiresistors. Along with the demands for better performance, novel approaches have been employed such as the formation of heterojunctions by combining more than one MOS [5,6,7], the addition of catalytic metal particles onto MOS (Pt, Pd, Rh, etc.) [8,9,10,11], MOS nanostructuring [12,13,14,15], and MOS doping or the formation of ternary MOS [16,17]. Ternary oxides such as Zn_2_SnO_4_ [18], ZnWO_4_ [16,19], NiWO_4_ [20], and CuWO_4_ [21,22] are attractive candidates for the research in this field. Copper tungstate (CuWO_4_) is a promising chemiresistor due its chemical and thermal stability and the presence of metal cations Cu^2+^ and W^6+^ providing adsorption sites [23,24,25]. Only a few research works have been performed on the combination of CuWO_4_ with other materials. For example, CuWO_4_/CuO heterojunctions were prepared using wet-based techniques [26,27,28]. However, these techniques limit the control of the purity and the composition of the final material. In our recent work [22], we investigated the combination of CuWO_4_/WO_3−*x*_ layers prepared by reactive sputtering, which is a deposition technique compatible with the thin-film industry and does not require a high temperature for synthesis.

With the design and development of novel MOS sensing materials, well-known cross-sensitivity to air humidity remains a crucial factor that needs to be addressed. Several strategies have been proposed and implemented to reduce the humidity interference in the sensing response [29,30,31,32]. Among others, one of the efficient strategies is the use of noble metal catalysts such as Pd, Pt, and their compounds [33,34,35].

In this work, we explore the possibility of synthesizing and using an MOS thin-film system based on CuWO_4_/CuO. This material system outperforms single-constituent materials regarding hydrogen sensing. Moreover, to address the issue of humidity influence, we added another layer formed by Pd particles and a nanostructured film of PdO. We show that this complex, three-layer system can be operated at lower temperatures and its response at various values of relative humidity can be equalized. To the best of our knowledge, this is the first work dealing with the humidity influence on the CuWO_4_-based system.

## 2. Materials and Methods

### 2.1. Material Synthesis

The investigated thin-film materials were prepared in the form of a layered structure by consecutive sputter depositions from metallic targets onto oxidized Si wafers (with several micrometers thick, thermally grown SiO_2_) with the dimensions of 8.5 × 8.5 × 0.6 mm^3^.

**Figure 1:**
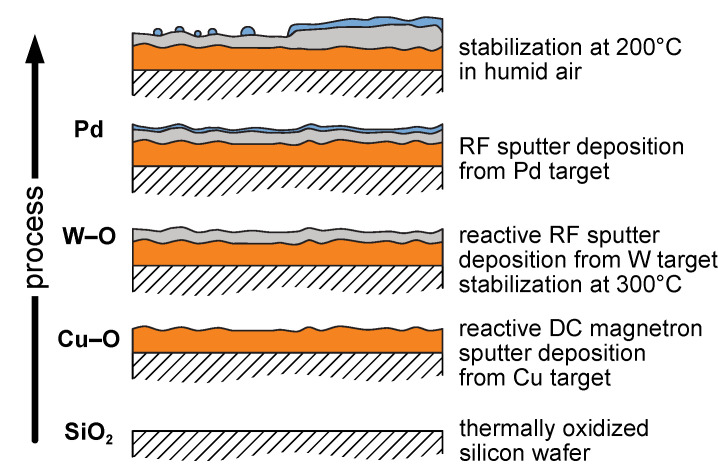
A scheme and description of the deposition process. The exact composition of individual layers is discussed below.

First, Cu–O layers were deposited in an Ar + O_2_ mixture by radio frequency (RF) sputtering using a Cu target (target diameter of 72 mm, target power of 228 W, Ar/O_2_ flow ratio of 2:1, substrate temperature of 400 °C, working pressure of 0.75 Pa at Ar flow of 10 sccm). The consecutive deposition of W–O layers was performed in an Ar + O_2_ mixture by direct current (DC) magnetron sputtering using a W target (target diameter of 72 mm, target power of 60 W, Ar/O_2_ ratio of 4:1, substrate temperature of 400 °C, working pressure of 0.60 Pa at Ar flow of 15 sccm). The conditions of the depositions were preoptimized to prepare crystalline Cu–O and W–O layers. Finally, the selected bilayers were then decorated with a small amount of palladium. Pd was deposited in an Ar gas by RF sputtering using a Pd target (target diameter of 50 mm, target power of 37.5 W, no external heating, working pressure of 0.50 Pa at Ar flow of 10 sccm). A schematic view of the film architecture formation during the deposition process is depicted in Figure 1.

All depositions were carried out in a multitarget stainless steel vacuum chamber (Z400, Leybold-Heraeus LH, Cologne, Germany) pumped by a turbomolecular pump backed with a scroll pump and a cold trap (cooled by liquid nitrogen). The target-to-substrate distance was 70 mm. The base pressure was always lower than 5 mPa.

As will be shown later, the materials were not synthesized in the form of distinct layers. Nevertheless, we find it appropriate to denote them by individual layers that are separated by a slash (/). The superscripts then indicate the thicknesses of the layers as they would be if deposited as single layers. For example, the film prepared by depositing 5 nm of tungsten oxide on the top of 20 nm copper oxide and finally decorated with 0.8 nm of Pd is labeled ^0.8^Pd/^5^W–O/^20^Cu–O. The dash (–) indicates that there could be more oxide phases present, even in the single layers.

### 2.2. Material Analyses and Sensing Measurements

To study the structural properties of the as-deposited films, X-ray diffraction (XRD) analysis was performed using a diffractometer (X’Pert PRO, PANanalytical, Malvern, UK) with a Cu K_α_ source of radiation in the Bragg–Brentano configuration. Further understanding of the crystalline phases was achieved by employing Raman spectroscopy (LABRAM HR Evolution, Horiba Jobin Yvon, Palaiseau, France) using a 532-nm laser.

Morphological analysis of the specimens was carried out with a scanning electron microscope (SEM) (SU-70, Horiba Ltd., Kyoto, Japan).

The XPS measurements were carried out in an ultrahigh vacuum chamber with a base pressure of 1.8 × 10^−8^ Pa using an energy analyzer (Phoibos 150, Specs GmbH, Berlin, Germany) in the large area lens mode with 20 eV pass energy. The spectra were recorded using an Al_Kα_ X-ray source with an energy of 1486.6 eV. The charging effect was corrected using the reference peak of C 1 s (284.6 eV).

The gas sensing response was measured by a four-point method using a custom-build system. The heated sample was maintained under the constant flow of synthetic air (dry or humidified). Near to the chamber with the specimen, hydrogen gas could be introduced by a flow controller, resulting in a concentration change. For further details, see Appendix A or our previous works in [11,22]. The samples were directly stabilized under the measurement conditions. The electrical resistance, as well as the response, were stabilized after a few hours. Such stabilized material is denoted as ‘measured’ in the following text.

For the purpose of this paper, we define the sensitivity for the *n*-type material as:

(1) $$S=\frac{R_{a}}{R_{g}},$$

where *R*_a_ and *R*_g_ are the resistances in the presence of synthetic air and H_2_ gas mixed with air, respectively. For the *p*-type, the reciprocal relation is used according to [36].

## 3. Results and Discussion

In this section, we describe the composition, structure, and surface morphology of the synthesized thin-film materials. Further, we move on to the description of the sensing behavior and differences between the prepared films. At the end, we discuss the influence of structural differences on sensing performance.

We prepared numerous combinations of layers, however, in this paper, we present only those that are interesting from the point of view of structure and/or sensing performance. Therefore, all films presented here are based on a 20 nm thick layer of Cu–O, which is then overlayered with a 5, 10, or 20 nm thick layer of W–O. After stabilization at 300 °C and a measurement of the sensing performance, the films were finally decorated with Pd and stabilized at 200 °C. Let us note that, for particular analyses, only key results are presented.

### 3.1. Structure

**Figure 2:**
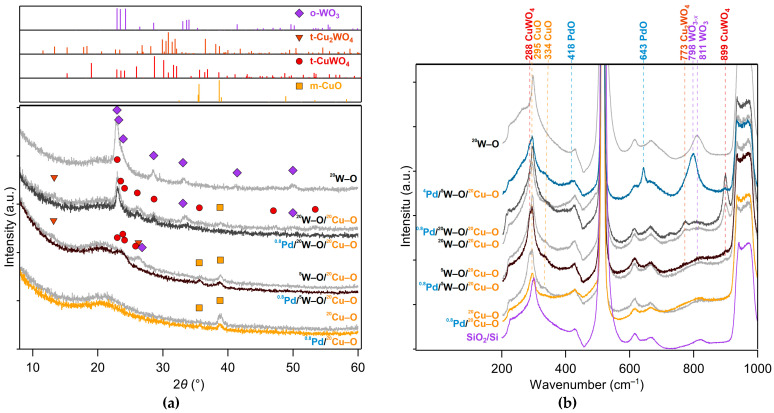
XRD spectrograms (**a**) and Raman spectra (**b**) of the measured films. The gray traces are Pd-free films while the color traces are the films decorated with ^0.8^Pd and measured, that is, stabilized at 200 °C. (Decoration with ^0.8^Pd itself does not change the structure.) The important Raman features are tagged by dashed lines with description of the position of the vibrational mode (in cm^−1^) and the corresponding compound. Most of the other features belong to silicon and/or silicon oxide, plotted in purple.

The structure of the films was studied by XRD and Raman spectroscopy. Figure 2a displays XRD patterns and Figure 2b presents Raman spectra of both as-deposited (grey traces) and measured films (color traces).

As seen in Figure 2a, the single layers of ^20^Cu–O and ^20^W–O exhibit the monoclinic CuO (PDF Card No. 04-007-1375) and orthorhombic WO_3_ (PDF Card No. 04-007-2425) phases, respectively. A broad peak in the range of 18–24° corresponds to an amorphous silica phase of the thermally oxidized Si.

The deposition of W–O species onto the CuO layer does not result in the formation of a distinct bilayer structure of CuO and WO_3_, but rather leads to their intermixing and the formation of copper tungstates. The triclinic CuWO_4_ (PDF Card No. 04-009-6293) and Cu_2_WO_4_ (PDF Card No. 04-041-0948) phases can be observed in the as-deposited W–O/^20^Cu–O films. The more W–O species are deposited, the more clearly the tungstate phases are identified in the structure of the as-deposited films, as observed when comparing XRD patterns for the ^5^W–O/^20^Cu–O and ^20^W–O/^20^Cu–O films. The intermixing of the pre-deposited CuO with the arriving W–O species occurs due to an energetic flux of the incoming species, which is also promoted by an elevated deposition temperature of 400 °C. Besides tungstates, CuO and WO_3_ are also detectable in the structure of the as-deposited films but as minor phases, especially for the ^20^W–O/^20^Cu–O film.

As we observed that the decoration of the films with a small amount of Pd had no effect on the XRD patterns, we present directly measured films with a 0.8 nm addition of Pd in Figure 2 (colored traces). As can be seen in Figure 2a, a sensing measurement performed at an elevated temperature leads to a further intermixing of the materials. This is accompanied by the disappearance of diffraction peaks from Cu_2_WO_4_ and lower intensities of diffraction peaks for CuO and WO_3_. The dominant phase after the sensing measurement is CuWO_4_.

**Figure 3:**
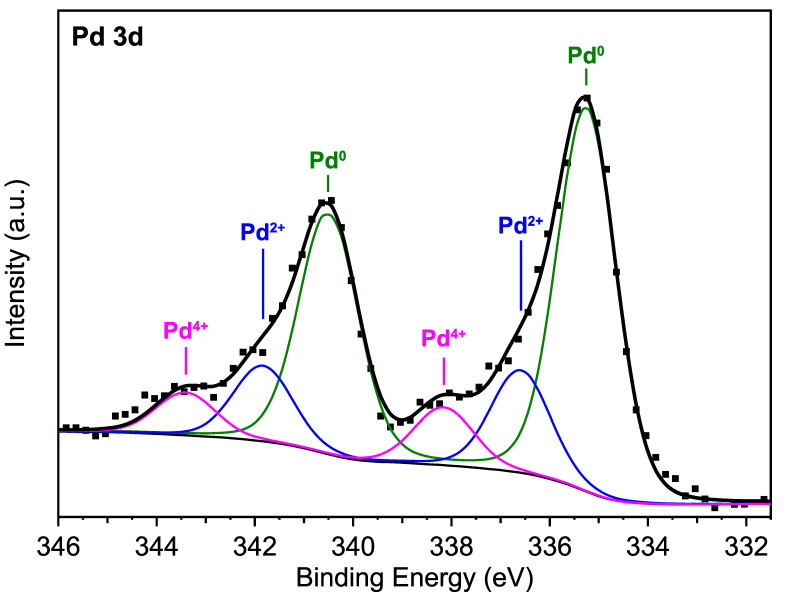
XPS spectra of the Pd 3d doublet of the ^0.8^Pd/^5^W–O/^20^Cu–O film. The best deconvolution consists of three components: metallic Pd^0^, Pd^2+^, and Pd^4+^ with 3d_5/2_ peak position at 335.3, 336.6, and 338.2, respectively. The 3d_3/2_ peaks were fitted at a fixed splitting energy of 5.25 eV.

The state of Pd is hard to identify only on the basis of XRD because of a negligible signal from really thin Pd-containing layers. However, we found that this can be partially overcome by using Raman spectroscopy when we analyzed a ^4^Pd-decorated film synthesized with the aim of increasing the signal of the Pd-containing layer. In Figure 2b, a Raman spectrum of this ^4^Pd/^5^W–O/^20^Cu–O film is shown. Peaks at the positions of 418 and 643 cm^−1^ [37,38] can be well associated with the occurrence of PdO. Using XPS, we also revealed that, after the deconvolution of the Pd 3d doublet (Figure 3), Pd is also present in another two states. The main component is attributed to the Pd^0^ state, while there are also minor Pd^2+^ and Pd^4+^ components present. The position of the oxidized states is in good agreement with the work of [39]. A slight shift of the Pd^0^ component to higher binding energies could be attributed to the small size of some Pd-containing particles (discussed later). This effect is described for evaporated Pd particles in [40].

Furthermore, Raman spectroscopy corroborates the results of XRD. Raman peaks observed in the as-deposited and measured multilayer films were identified by comparing them with the literature data and spectra of the single ^20^Cu–O and ^20^W–O films and of the thermally oxidized Si substrate. The peaks at 295 and 334 cm^−1^ are attributed to CuO [26,41] and can be found in the spectra of all multilayer films. However, when the amount of W–O is increased from ^5^W–O to ^20^W–O, the signal from CuO is reduced while the peak corresponding to CuWO_4_ at the position of 899 cm^−1^ [42,43] distinctly gains in its intensity. This confirms a nearly complete intermixing of W–O with Cu–O. The other phase of tungstate (Cu_2_WO_4_) was also observed at the position of 773 cm^−1^ [43,44] but with a much lower peak intensity. Other distinguishable peaks at the positions of 300, 428, 513, 615, 664, 821 cm^−1^, and a broad feature at 936–974 cm^−1^ are attributed to the SiO_2_/Si substrate.

### 3.2. Surface Morphology

**Figure 4:**
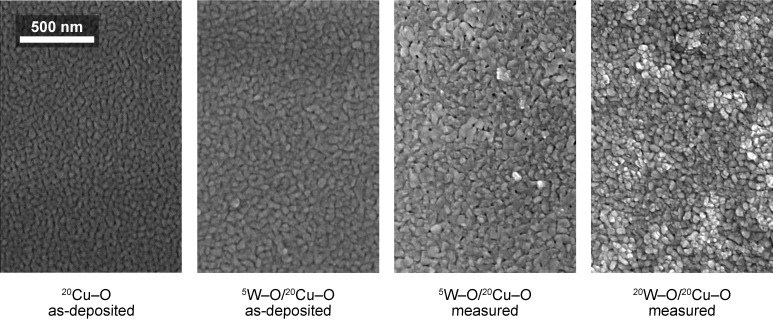
SEM micrographs of the as-deposited (**a**,**b**) and measured films (**c**,**d**) (at 300 °C in both dry and humid synthetic air). The brighter zones, possibly formed by tungstate, are visible in (**c**,**d**).

**Figure A2:**
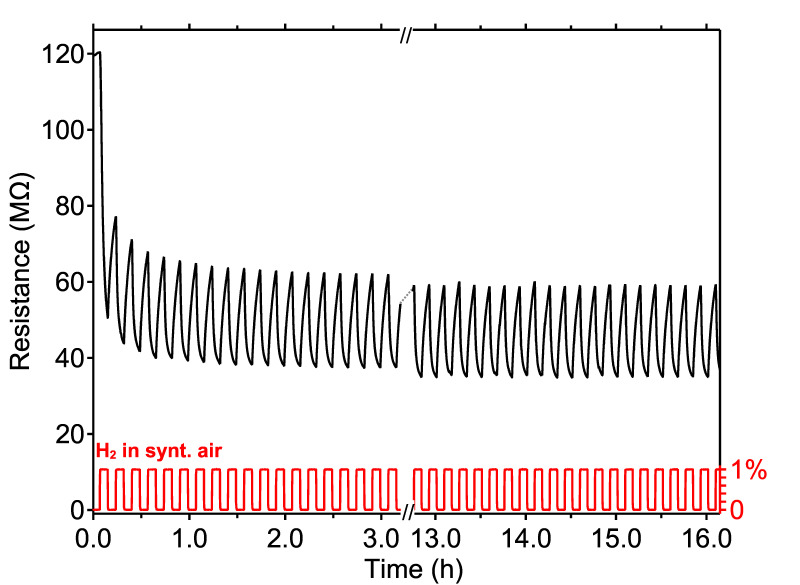
Record of ^20^W–O/^20^Cu–O stabilization curve. The hydrogen concentration was cycled for several hours to obtain a stable response. Then, the characteristics presented in text were recorded. The behavior of other specimens was similar.

The SEM micrographs in Figure 4 illustrate the evolution of the surface morphology with the overlayering of ^20^Cu–O by W–O in the as-deposited state and after the sensing measurements (stabilization) at 300 °C in both the dry and humid air. An example of the stabilization curve can be found in Figure A2 in Appendix B. The surface morphology of a single ^20^Cu–O layer in the as-deposited state in Figure 4a is of uniform contrast and the surface consists of irregular, fine features typical of sputtered films. The overlayering of this layer by ^5^W–O only results in an increase in the size of the surface features (Figure 4b). The sensing measurement at 300 °C, however, causes changes in the surface morphology. The surface is then characterized by zones of different contrasts (Figure 4c). This effect is more pronounced for the ^20^W–O/^20^Cu–O film (Figure 4d). In agreement with our previously published work [22] where an inverse material combination (^10^Cu–O/^20^W–O) was synthesized, and respecting the results of Raman spectroscopy and XRD, the brighter zones can be attributed to the formation of copper tungstate.

**Figure 5:**
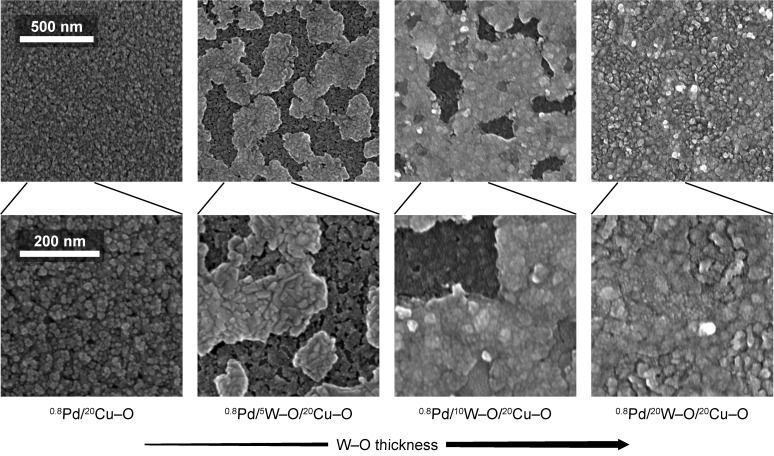
SEM micrographs of measured films decorated with palladium. A lower magnification (top row) reveals the evolution of the overall coverage of the film with the topmost Pd-containing layer. The morphology of this layer is well-imaged in a higher magnification (bottom row). The Pd forms oxidized small particles (well-visible in (**b**)) and bright zones on top of base bilayer (**b**–**d**). Not decorated films (**a**) are shown for a comparison.

**Figure 6:**
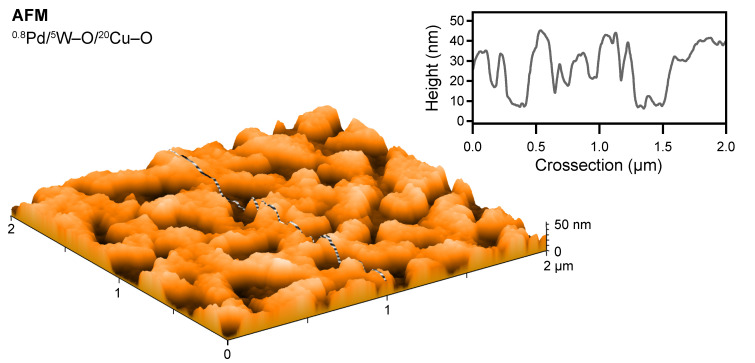
AFM image and cross-section of measured ^0.8^Pd/^5^W–O/^20^Cu–O film. The height of features covered with PdO ranges from approximately 20 to 30 nm.

The evolution of the zones of a different contrast is demonstrated by SEM micrographs in Figure 5. Here, films after decoration with ^0.8^Pd and the subsequent sensing measurements (and the stabilization) at 200 °C in both the dry and humid air are depicted. As shown in Figure 5a, the addition of Pd uniformly covers the single ^20^Cu–O layer and the sensing measurement does not initiate any pronounced changes in the surface morphology. However, a different situation is observed after the sensing measurements of the ^0.8^Pd/W–O/^20^Cu–O (Figure 5b–d). The surface of all these films is characterized by two different zones. The darker one is like that observed on the single ^20^Cu–O film (Figure 5a), while the brighter one is represented by islands that occupy more area with an increasing amount of added W–O (Figure 5b–d). Furthermore, the surface of the islands is raised above the dark zone level, as observed by AFM and shown in Figure 6 for the ^0.8^Pd/^5^W–O/^20^Cu–O film. We suppose that, as the films are subjected to further sensing measurement, i.e., annealed at 200 °C for several hours in humid air, the process of intermixing W–O with Cu–O continues, resulting in the formation of the islands of CuWO_4_ that grow in size and height. For the film with the highest amount of W–O (^0.8^Pd/^20^W–O/^20^Cu–O), the surface is nearly completely covered with CuWO_4_.

As Raman spectroscopy, and XPS especially, have shown, Pd oxidizes during the sensing measurement. While on the Cu–O surface it forms nanometer-sized particles (small white dots visible in Figure 5), the CuWO_4_ surface favors the formation of PdO [45] as a continuous layer. It is worth noting that the approximately 30 nm height of the islands (Figure 6) excludes these islands from being formed by PdO because there is simply not enough material for it. Interestingly, the appearance of the Pd particles in the dark zone is similar to that observed on ^2^Pd/^100^W–O films synthesized at similar parameters (published in our previous work [17]).

In summary, the consecutive deposition of the Cu–O and W–O layers does not lead to the formation of a distinct bilayer structure of binary oxides but leads to the formation of a bilayer of copper tungstate and cupric oxide, or of copper tungstate alone (depending on the amount of Cu–O). The decoration with Pd and the subsequent sensing measurement results in the formation of the CuWO_4_ islands that are covered with the PdO film and the nanoparticles of PdO on the Cu–O surface. According to other works [46,47], it is reasonable to presume that the Pd particles are of a core-shell nature (a Pd core covered by PdO), yet XRD or Raman spectroscopy are not capable of confirming this.

### 3.3. Sensing Response

The synthesized films were stabilized in the measuring chamber at elevated temperatures under the measuring conditions (300 °C for the Pd-free films and 200 °C for the Pd-decorated films). After several hours of stabilization, the response curves were recorded and the films were exposed to various concentrations of H_2_ in synthetic air. First, we demonstrate how the bilayer W–O/^20^Cu–O system outperforms the response of the single-layer ^20^CuO film. Then, we explain how the humidity in the air negatively affects the response and how this unwanted effect can be reduced by decorating the films with Pd and by tuning the working temperature.

**Figure 7:**
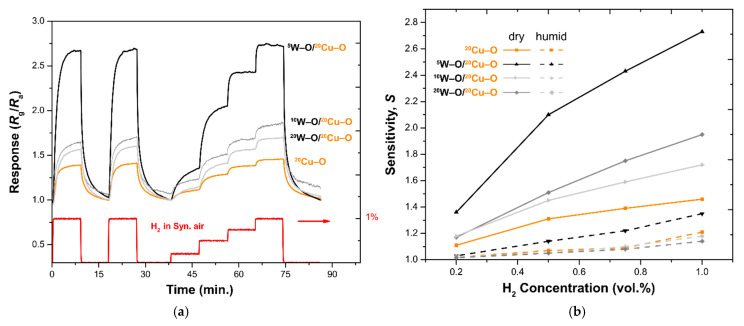
(**a**) Response of synthesized films without decoration with Pd. The combination of layers enhances the response to the hydrogen. (**b**) Sensitivity of the same films. Solid lines represent the sensitivity in dry air, dashed lines for humid air (90% RH). The films with W–O exhibit a better sensitivity than ^20^Cu–O alone; the highest sensitivity is for ^5^W–O/^20^Cu–O.

**Figure 8:**
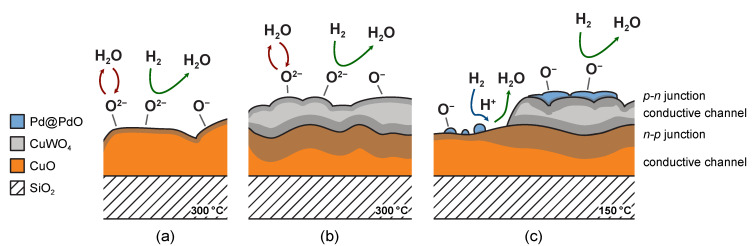
Scheme of synthesized structures with indicated reactions on the surface of (**a**) single Cu–O film, (**b**) W–O/Cu–O bilater (**b**) and (**c**) Pd/W–O/Cu–O trilayer. Detailed explanation in text.

Response curves (measured in dry air at 300 °C) of the Pd-free films are illustrated in Figure 7a, where the response *R*_g_/*R*_a_ vs. time is plotted. The yellow curve shows the behavior of the single-layer ^20^CuO film (S~1.4). The response of this film is enhanced nearly twice (S~2.7) if ^5^W–O is deposited on the top (^5^W–O/^20^Cu–O). However, a further addition of W–O (^20^W–O/^20^Cu–O) results in a decrease in the magnitude of the response, although this is still better than for ^20^CuO (S~1.4) and CuWO_4_ (S~1.2) alone. Enhancement for the ^5^W–O/^20^Cu–O film can be explained by the formation of CuWO_4_ over the CuO layer, leading to the development of n-p heterojunctions. A depletion layer is formed between n-type CuWO_4_ and p-type CuO. The situation is depicted in Figure 8a,b. Due to this, the conductive channel is reduced for the film and so the surface reactions with hydrogen cause a bigger change in the resistance. This is also in agreement with the shift of the resistance baseline from 300 kΩ to 550 kΩ for ^20^Cu–O to ^5^W–O/^20^Cu–O, respectively. A complete overview of all specimens’ baseline resistances can be found in Table S1 in the Supplementary Materials. The trend and explanation are found to be similar to the previously studied structures where CuWO_4_ was grown on the top of a W–O layer forming n-n junctions [22] and is further documented in other works for similar materials [46,47].

The numerically calculated values of sensitivity according to Equation (1) for the described bilayers are plotted in Figure 7b. The sensitivity values for various concentrations of H_2_ in dry air are represented by solid lines, while the dashed lines correspond to the sensing measurements in humid air.

Sensitivity decreases in the humid environment; the effect of reduced sensitivity in humid air is described in the literature [31,48]. The interference of humidity negatively affects the gas-sensing behavior of materials, primarily for reducing gases such as H_2_ and H_2_S. A discussion of this effect follows.

In the dry condition, the sensing mechanism can be described by the following equations [49,50,51] (green arrows in Figure 8):

(2) $$\frac{1}{2}O_{2}+2e^{-}\to O_{\mathrm{ads}}^{2-}$$

(3) $$H_{2\;\mathrm{ads}}+O_{\mathrm{ads}}^{2-}\to H_{2}O+2e^{-}$$

However, when the bilayers are exposed to gas in a humid environment, the water molecules react with adsorbed oxygen species on the surface, leading to an increase in the baseline resistance of the bilayer and a decrease in its sensitivity. At the same time, hydrogen cannot find the oxygen species to react with and the interchange of electrons, and thus the response is reduced [31,52] (red arrows in Figure 8).

The adsorption of water can be described by one of the two following mechanisms as

(4) $$H_{2}O+M+O_{O}\to \left(M^{+}–{\mathrm{OH}}^{-}\right)+{\left(\mathrm{OH}\right)}_{O}^{+}+e^{-}$$

or

(5) $$H_{2}O+2M+O_{O}\to 2\left(M^{+}–{\mathrm{OH}}^{-}\right)+V_{O}^{2+}+2e^{-}.$$

Both involve the formation of OH^–^ groups on the surface that are bonded to the metal ion M^+^. According to the first mechanism, Equation (4), the other hydrogen from the water molecule forms a rooted hydroxyl group ${\left(\mathrm{OH}\right)}_{O}^{+}$ with the lattice oxygen $O_{O}$. In the other proposed mechanism, Equation (5), the lattice oxygen forms another adsorbed OH^–^ bonded to M^+^ while leaving an oxygen vacancy $V_{O}^{2+}$.

To address the issue regarding the reduction in the response, the films were decorated with 0.8 nm of Pd. According to Raman spectroscopy, XRD, and other published works on similar systems [47,53], Pd particles/objects are covered with PdO on their surfaces. Pd and PdO play several important roles in the reaction (see also Figure 8c):

(i) Pd promotes the selectivity towards hydrogen and, at the same time, reduces the response temperature by facilitating the dissociation of H_2_ [46] (blue arrow in Figure 8c).
(ii) PdO favors the adsorption of oxygen over the hydroxyl groups [53].
(iii) PdO forms another heterojunction (p–n) with the topmost layer of copper tungstate.

**Figure 9:**
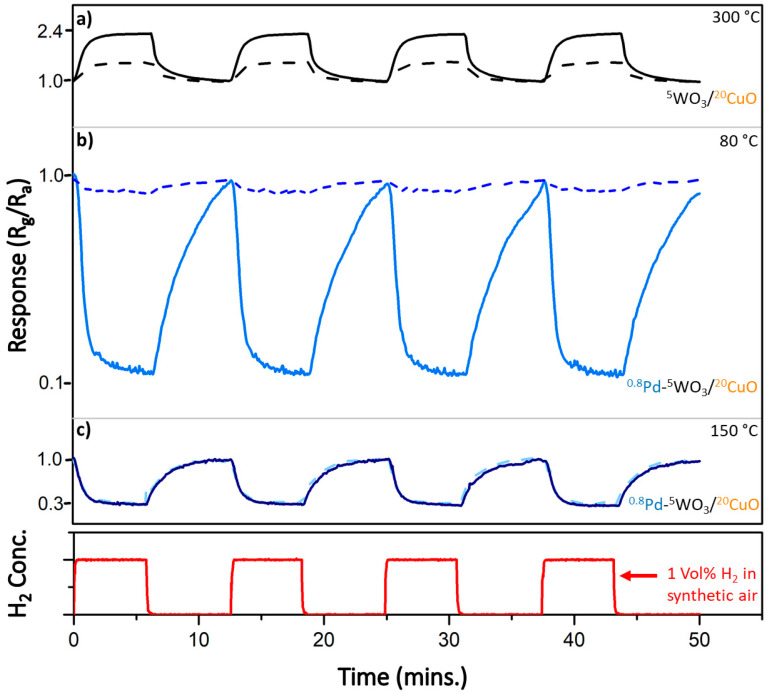
Sensing response for Pd-decorated film (^0.8^Pd/^5^W–O/^20^Cu–O) (**b**,**c**) and Pd-free film (**a**). The response at lower temperatures switches to n-type semiconductor. The solid lines represent response in dry air, dashed lines for humid air (96% RH).

Furthermore, the presence of Pd allowed us to reduce the response temperature and stabilize the films at only 200 °C. After stabilization, the response of the films was measured at various lower temperatures. The response curve in Figure 9a is of the Pd-free film (^5^W–O/^20^Cu–O) measured at a temperature of 300 °C, where the sensitivity was the highest. In Figure 9b, the response of the Pd-decorated film (^0.8^Pd/^5^W–O/^20^Cu–O) measured at 80 °C only is shown. At this temperature, the sensitivity was the highest (~8.2). On the other hand, the effect of humidity is enormous and the responses in humid and dry conditions differ greatly; compare solid lines (dry air) and dashed lines (humid air, 90% RH). When the operating temperature was adjusted, we were able to equalize the response to hydrogen in dry and humid air. At 150 °C, the sensitivity is partially reduced (~3.4), but the response is the same regardless of humidity (Figure 9c). This can be explained by the fact that the OH groups on the surface desorb at higher temperatures, which can partly eliminate the humidity effect [53,54]. At these conditions, the minimum detectable concentration of hydrogen was 500 ppm.

It is important to note that the character of the response changes when moving to the lower temperatures. The film responds as n-type (the resistance decreases with hydrogen) at lower temperatures (80 °C and 150 °C), while it behaves as p-type at 300 °C. This can be explained by the layered architecture of the film. As the different types of semiconductors are synthesized on top of each other, they can be imagined as parallelly connected resistors. At higher temperatures, the reaction on p-type CuO dominates. At lower temperatures, CuO does not respond at all, or its contribution is minor, and the resistance change in n-type CuWO_4_ dominates.

**Figure 10:**
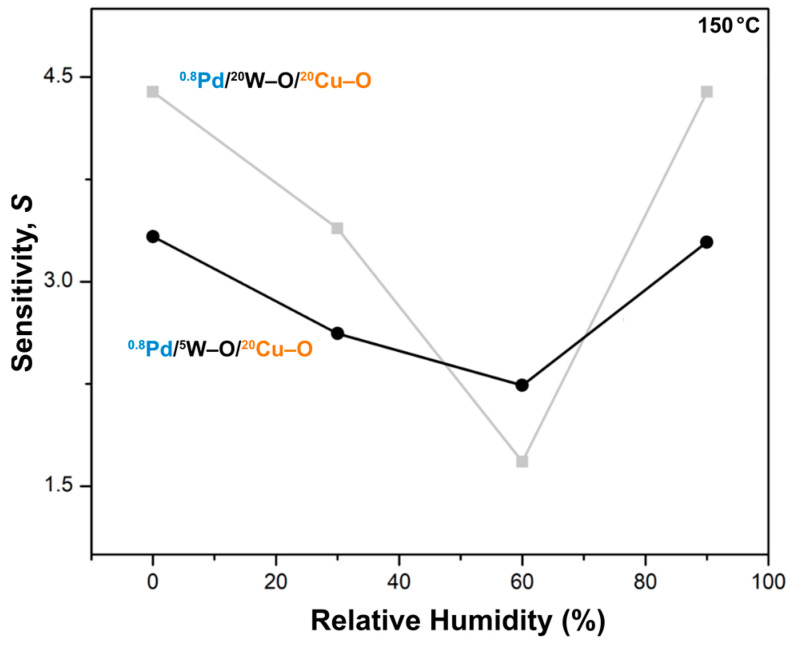
Sensitivities of Pd-decorated films at low temperature. The film with more pronounced multilayer structure (^0.8^Pd–^5^W–O/^20^Cu–O) exhibits more equalized response to hydrogen under various relative humidity levels.

Finally, let us explain why it is important to employ both films and not only use a single-phase film decorated with Pd. The plot in Figure 10 shows the response of the two ^0.8^Pd/^5^W–O/^20^Cu–O and ^0.8^Pd/^20^W–O/^20^Cu–O films to 1 vol.% of H_2_ at various levels of humidity. According to the previous section, the architecture of the first film is copper tungstate on top of CuO. The other film is mainly formed by a copper tungstate (with only a negligible remaining layer of CuO). The response trend is found to be similar for both films, but for the ^0.8^Pd/^5^W–O/^20^Cu–O film it is more stable over the humidity scale. Humidity shifts the baseline of the resistance of the materials. The ^0.8^Pd/^5^W–O/^20^Cu–O film exhibits more humidity-independent sensitivity because, even if CuO does not notably contribute to the response to H_2_, it compensates for the effect of humidity in the other direction. We believe that this is mainly because of the narrowed conduction channel formed by the n-p heterojunctions in-between CuWO_4_ and CuO. Due to this, the response at various levels of humidity is equalized.

A comparison of the sensitivity parameters with other works is provided in Table 1. Here, a few bilayer materials containing CuO, as well as systems with Pd, are listed. Most of them show a better sensitivity to hydrogen but at higher working temperatures. The influence of humidity is not generally comparable (an equalized response can be found, for example, in work [55] on the Pd–CuO system). A further enhancement of the sensing performance of the three-layer system presented here could be possible by a more precise tuning of the thickness ratios of constituent layers as well. A more thorough understanding of the formation of Pd-containing zones will be necessary to elaborate and optimize the presented MOS thin-film system.

## 4. Conclusions

We demonstrated a synthesis of a thin-film material consisting of three types of layers: cupric oxide, copper tungstate, and palladium oxide particles/film. The synthesis process consisted of three consecutive steps: reactive sputtering from the copper target, reactive magnetron sputtering from the tungstate target, and sputtering of palladium. The stabilization of the films during the sensing measurements in dry and/or humid air was also crucial for the formation of individual phases. The structure and the surface morphology of the films were then described in detail.

The measurement of the conductometric response to hydrogen showed that the response of cupric oxide alone can be enhanced by depositing tungsten oxide on the top, which leads to an intermixing of both phases. As a result, a layer of copper tungstate was formed along with n-p heterojunctions in-between the top *n*-type CuWO_4_ layer and the bottom *p*-type CuO layer. These bilayers suffer from enormous interference of the relative humidity when detecting the hydrogen gas. This issue was resolved by adding Pd. After stabilization at 200 °C, a third layer consisting of a PdO film and nano-sized, oxidized Pd particles was formed. Consequently, the response in dry and humid air was equalized at only 150 °C. This could be explained by a mutual compensation of the changes in humid environment by the n-p heterojunctions in-between CuWO_4_ and CuO.

This complex material is a good candidate for use in conductometric sensors. The relatively simple synthesis is compatible with thin-film technology and does not require high temperatures.

## Figures and Tables

**Table 1:** An overview of similar material systems used for hydrogen detection. Due to the different definitions of sensitivity, the relevant definition is included.

Sensing Material and Morphology		Sensitivity *S*		Temperature (°C)	Concentration (ppm)	References	
		Value (1)	Definition				
Pd/CuWO_4_/CuO		8.2 (Dry) 3.4 (Dry) 3.4 (Humid)	*R*_a_/*R*_g_	80 150 150	10,000	This work	
CuO	CuO film	1.25	R_H2_/R_a_	400	125	A. Rydosz et al. (2018)	[56]
CuO/TiO_2−y_	CuO/TiO_2_ films	1.5	R_H2_/R_a_	400	125	A. Rydosz et al. (2018)	[56]
Pd/CuO	Pd-decorated CuO nanorods	4.5	(*I_a_* − *I_g_)/I_g_*	200	1000	N. Sarıca et al. (2019)	[57]
CuWO_4_/WO_3_	CuWO_4_/WO_3_ heterojunction	5.0	(R_a_ − R_g_)/R_g_	350	10,000	Kumar et al. (2020)	[22]
Zn/CuO	Zn-doped CuO	7 (Dry) 3.8 (Humid)	(R_g_ − R_a_)/R_a_	650	100	V. Cretu et al. (2016)	[58]
CuO/WO_3_	CuO nanoclusters on WO_3_	2.4	(R_a_ − R_g_)/R_g_	300	10,000	S. Haviar et al. (2018)	[11]
CuO/ZnO	CuO/ZnO heterocontacts	2.3	I_H2_/I_a_	400	4000	S. Aygün et at. (2005)	[59]
CuO/TiO_2_	CuO/TiO_2_ nanocomposites	2–3	(R_g_ − R_a_)/R_a_	200	1000	D. Barreca et al. (2011)	[60]
Pd/CuO	Pd-capped CuO thin film	3.8 (Dry) 3.0 (Humid)	R_a_/R_g_	300	1000	P. Yadav et al. (2020)	[55]

**Figure A1:**
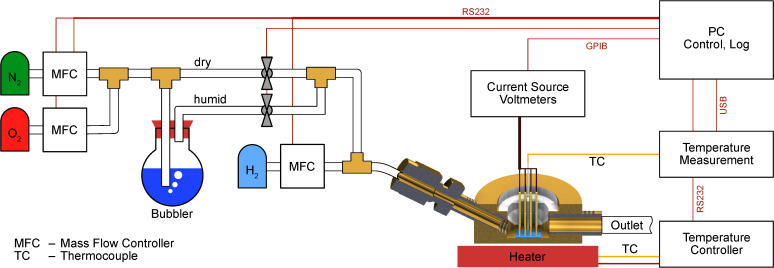
A scheme of an experimental system for the testing of the sensorial response. The overall process is driven by a LabVIEW-based software run on the computer. The electrical contact to the measured material is provided by spring-loaded, gold-plated tips linearly aligned with a pitch of 2 mm.

## Data Availability

Data can be available upon request from the authors.

## Supplementary Materials

The following are available online at https://www.mdpi.com/article/10.3390/nano11123456/s1, Table S1: Baseline resistances of investigated sensing films.

## Appendix A

### Gas Sensing Testing System Description

The gas response was measured using a four-point probe measurement technique in flow mode using a custom-built, heated brass reaction chamber (total volume: 9 cm^3^) with gas inlet and outlet slots. Synthetic air was mixed from clean gases (N_2_, O_2_) in a 79:21 ratio using two flow controllers (Alicat Scientific Ltd., Tucson, AZ, USA). The tunable portion of the synthetic air can be humidified by bubbling through a warm water bath and then mixing again with the dry portion of the air. The resulting relative humidity is measured in the tube using a sensor (SHT 85, Sensirion, Staefa, ZH, Switzerland). Close to the measuring chamber, hydrogen is introduced into the flow using another flow controller, enabling to tune its final concentration. The total flow is maintained at 100 sccm. Further details about the system can be found in our previous works in [11,22] or in the scheme of the system in Figure A1.
