# Supplementary material for: Three-Layer PdO/CuWO4/CuO System for Hydrogen Gas Sensing with Reduced Humidity Interference
Source: Nanomaterials (Basel). 2021 Dec 20;11(12):3456. doi: 10.3390/nano11123456 (PMC8704960; doi:10.3390/nano11123456)
Supplement: Supplementary file 1 [file nanomaterials-11-03456-s001.zip › nanomaterials-1479532-supplementary.pdf]

nanomaterials

MDPI

Supplementary Material

Three-Layer PdO/$CuWO_4$/CuO System for Hydrogen Gas Sensing with Reduced Humidity Interference

Nirmal Kumar, Stanislav Haviar * and Petr Zeman

Department of Physics and NTIS—European Centre of Excellence, Faculty of Applied Sciences, University of West Bohemia, Plzeň 301 00, Czech Republic; kumarn@kfy.zcu.cz (N.K.); zemanp@kfy.zcu.cz (P.Z.)
* Correspondence: haviar@ntis.zcu.cz; Tel.: +420-377-632-220

Table S1. Baseline resistances of investigated sensing films.

Specimen	Temperature	Dry Baseline Resistance	Humid Baseline Resistance
^{20}Cu–O	300 °C	0.3 MΩ	0.3 MΩ
^{5}W–O/^{20}Cu–O	300 °C	0.5 MΩ	2.0 MΩ
^{10}W–O/^{20}Cu–O	300 °C	0.7 MΩ	1.5 MΩ
^{20}W–O/^{20}Cu–O	300 °C	1.3 MΩ	2.4 MΩ
$^{0.8}$Pd/^{20}Cu–O	100 °C	0.8 GΩ	1.2 GΩ
$^{0.8}$Pd/^{5}W–O/^{20}Cu–O	100 °C	0.6 GΩ	2.6 GΩ
$^{0.8}$Pd/^{10}W–O/^{20}Cu–O	100 °C	3.2 GΩ	4.8 GΩ
$^{0.8}$Pd/^{20}W–O/^{20}Cu–O	100 °C	50 MΩ	40 MΩ

Specimen	Humidity	Sensitivity Dry	Humid Baseline Resistance
$^{0.8}$Pd/^{5}W–O/^{20}Cu–O	0	3.33	5 GΩ
	30	2.62	6 GΩ
	60	2.24	4 GΩ
	90	2.21	8 GΩ
$^{0.8}$Pd/^{20}W–O/^{20}Cu–O	0	4.39	44 MΩ
	30	3.39	44 MΩ
	60	1.68	46 MΩ
	90	1.70	45 MΩ
